# Preparation of sodium alginate-coated microencapsulated phage vB_SalP_SE29 and evaluation of its therapeutic effect on *Salmonella* enteritidis infection

**DOI:** 10.1128/aem.02263-25

**Published:** 2026-02-27

**Authors:** Yan Cheng, Jiahao Yu, Xiaochen Ren, Ruiqi Liang, Bingmei Du, Jinhong Li, Sayed Haidar Abbas Raza, Wuwen Sun, Dongxing Zhang, Lei Zhang

**Affiliations:** 1 College of Animal Science and Technology, College of veterinary Medicine, Jilin Agricultural University 85112, Changchun, China; 2 Guangdong Provincial Key Laboratory of Food Quality and Safety, South China Agricultural University, Guangzhou, China; 3 State Key Laboratory of Biocontrol, School of Life Sciences, Sun Yat-Sen University, Guangzhou, Guangdong, China; 4 Borui Technology Co., Ltd. 12526, Changchun, China; University of Nebraska-Lincoln, Lincoln, Nebraska, USA

**Keywords:** phage, microencapsulation, therapeutic, *Salmonella*, sodium alginate

## Abstract

Phages have demonstrated significant therapeutic efficacy in treating infections caused by antibiotic-resistant bacteria, yet their poor stability in the gastrointestinal tract limits oral application. This study aimed to address this barrier by preparing microencapsulated phage vB_SalP_SE29 (a lytic phage against *Salmonella*) using the droplet method, with gelatin, sodium alginate, and calcium chloride as wall materials. We optimized the microencapsulation process via single-factor experiments and orthogonal design, then evaluated the microspheres’ biological properties and *in vivo* therapeutic efficacy in a rat model of *Salmonella* enteritis. The optimal formulation (2% sodium alginate, 8% gelatin, and 1.65% calcium chloride) achieved an encapsulation efficiency of 86.52% and a titer of 1 × 10^7^ PFU/mL. The potency remained stable in simulated gastric fluid at pH 2, was stable in 1% and 2% bile hydrochloric acid, and displayed improved release in simulated intestinal fluid. Storage at 4°C for 6 weeks reduced microencapsulated phage titer by 0.146 lg PFU/g (vs. 0.591 lg PFU/mL for free phages). Furthermore, *in vivo*, microencapsulated phages increased the survival rate of infected rats to 70% (vs. 40% for free phages). Microencapsulated phage also significantly reduced inflammatory factors compared to free phage and notably improved organ lesions in infected rats. These findings demonstrated that gelatin-sodium alginate-calcium chloride microencapsulation enhanced phage stability and therapeutic efficacy, supporting the potential of microencapsulated vB_SalP_SE29 as a safe bio-antimicrobial for combating *Salmonella* infections.

**IMPORTANCE:**

*Salmonella* is a common gram-negative bacterium widely distributed in nature, as well as in the intestines of humans and animals, and serves as a typical representative of foodborne pathogens. Due to the increasingly prominent issue of its multidrug resistance, phage therapy has garnered extensive attention as a potential alternative. However, when administered orally, phages are readily inactivated by gastric acid, leading to a significant reduction in phage titer. As a biopolymer with excellent biocompatibility, low cost, and low toxicity, sodium alginate can form a gel through cross-linking with Ca²⁺ ions at room temperature. The preparation of microencapsulated phages effectively protects phages from gastric acid damage, thereby enhancing their antibacterial efficacy. Evaluation of relevant biological characteristics has demonstrated that microencapsulated phages significantly improve their survival ability in the gastrointestinal environment. *In vivo* studies have further confirmed the good efficacy and safety of these microencapsulated phages in the treatment of rat enteritis.

## INTRODUCTION

*Salmonella*, a member of the *Enterobacteriaceae* family, is widely distributed in the natural environment and can be found in food, water, and the intestinal tract of various animals (1). It has been a common pathogen in the aquaculture industry, causing diseases such as septicemia, enteritis, and diarrhea in livestock and poultry and posing a significant threat to public health (2). Previously, the main biological control measures relied on the use of antibiotics. However, due to the widespread and irrational use of antibiotics, there has been an emergence of drug residues and drug-resistant bacteria, leading researchers to gradually shift their focus to the research and development of alternative antibiotic products (3).

Phages, which are ubiquitous in natural environments and exhibit highly specific bacterial targeting capabilities, possess several unique therapeutic advantages. Their narrow host specificity prevents disruption of normal gut microbiota (unlike broad-spectrum antibiotics). They pose minimal risk of inducing bacterial resistance due to co-evolutionary dynamics with bacteria. They are capable of self-replication at infection sites as long as susceptible host bacteria are present, thereby amplifying their antibacterial effects over time; however, they are unable to persist in the absence of their bacterial hosts. They demonstrated low cytotoxicity toward eukaryotic cells, ensuring excellent host safety. These combined characteristics position phage therapy as a particularly promising treatment modality for bacterial infections (4). Due to their ability to lyse their bacterial hosts, lytic phages have been applied in a wide variety of scenarios to combat bacterial pathogens, including therapy (as therapeutic and prophylactic), biocontrol, sanitation, preservation, detection, and remediation (4, 5). Phages are widely used in the treatment of freshwater fish, livestock, and poultry. For example, they can be used to treat the pathogenic strains of *Vibrio harveyi* in fish, reduce persistent *Salmonella* and *Campylobacter* in poultry, and control *Salmonella* in the peripheral lymph nodes of cattle (6–8). As an effective supplementary method to antibiotic therapy, phages are increasingly recognized for clinical anti-infection treatments in more and more countries and regions. Therefore, there is an urgent need for systematic and in-depth research to promote the standardization and safety of phage clinical applications.

Phages exhibit poor resistance to acidic and alkaline conditions and are easily inactivated during phage therapy, thereby hindering their therapeutic efficacy (9). Consequently, microencapsulation is necessary for enhancing their stability. Microencapsulation technology involves using natural or synthetic polymer materials to encapsulate or disperse solids, liquids, or gases within a carrier matrix, resulting in the formation of microparticles. Microencapsulated phages can significantly improve resistance to acidic and alkaline environments, as well as enhance stability in gastric fluid, bile salts (BS), and intestinal fluid, along with their stability during storage. Although microencapsulation offers numerous advantages, it is crucial to select appropriate coating materials and preparation methods to ensure the phages’ viability and maximize their therapeutic potential. As a result, numerous researchers are exploring alternative delivery methods such as nanoparticles, liposomes, spray drying, or microspheres to address this challenge (9–11). Colom administered liposome-encapsulated phages through drinking water and feed to treat broilers infected with *Salmonella* spp. (12). Abdelsattar utilized phage microencapsulation for targeted delivery to intestinal bacteria in livestock (13). Islam employed encapsulation techniques, including spray drying and extrusion methods, to safeguard phages from harsh intestinal conditions (14). However, many existing encapsulation systems face limitations such as moderate encapsulation efficiency, complex preparation processes, high cost, or insufficient stability under the extreme acidic conditions of the stomach. Therefore, there is a clear need to develop a simple, cost-effective, and highly protective oral delivery platform that can maintain high phage viability throughout the gastrointestinal tract while enabling controlled release in the intestine.

In the current study, we encapsulated phages into microencapsulated spheres using sodium alginate, gelatin, and calcium chloride to treat enteritis in rats caused by *Salmonella* S.E-29. According to our findings, there has been limited research on phage encapsulation using sodium alginate, gelatin, and calcium chloride. The main objective of this study was to investigate the biological properties of microencapsulated phages encapsulated with these materials. As a proof of concept, we verified and analyzed the therapeutic effects through survival rate analysis, inflammatory factor assays, and pathological section examinations. This study provides a theoretical basis for the preparation and application of microencapsulated phages.

## MATERIALS AND METHODS

### Rats

Female Sprague-Dawley (SD) rats (weighing 200 ± 20 g) were purchased from Liaoning Changsheng Biotechnology Co., Ltd. (Liaoning, China; Certificate No.: SCXK[Liao]2020-0001) and housed in a specific-pathogen-free environment at Jilin Agricultural University. These rats were maintained under a 12 h light and 12 h dark cycle, with the temperature kept at 25°C ± 3°C.

### Materials

Sodium alginate ([C_6_H_7_O_6_Na]_*n*_; Solarbio, China), gelatin (Solarbio, China), and calcium chloride (Macklin, China) had a purity of 96% and a molecular weight of Mw = 110.88 kDa (data provided by the supplier).

### Strains and phage

*Salmonella* S.E-29 was isolated, identified, and stored in our laboratory. This strain needed to be cultured at 37°C in Luria Bertani (LB, peptone [Oxoid, UK] 1 g; yeast extract [Oxoid, UK] 0.5 g; NaCl 1 g; sterile water to make up to 100 mL) medium. Phage vB_SalP_SE29 was isolated from a sewage system in Changchun, China, and stored in our laboratory. Phage isolation, plaque assays, and spot tests were performed according to previously reported methods (15).

### Biological characteristics of vB_SalP_SE29 phage

Methods described in previous literature were adapted and optimized (16, 17). *Salmonella* S.E-29 was inoculated into LB liquid medium and cultured at 37°C with shaking at 120 rpm until the optical density at 600 nm (OD₆₀₀) reached 0.6–0.8. To evaluate the *in vitro* bactericidal activity of phage vB_SalP_SE29, OD₆₀₀ values were measured at eight time points: 0, 20, 40, 60, 80, 100, 120, and 140 min. For the determination of the optimal multiplicity of infection (MOI), *Salmonella* S.E-29 cultures were mixed with the phage at ratios of phage-to-host bacteria of 100, 10, 1, 0.1, 0.01, and 0.001, transferred to LB medium, and incubated at 37°C for 6 h. To construct the one-step growth curve of vB_SalP_SE29, an appropriate volume of phage was adsorbed to host bacteria at 37°C for 15 min according to the pre-determined optimal MOI. Unadsorbed phages were removed by centrifugation, and the bacterial pellet was resuspended in LB medium. Samples were collected every 10 min for plaque-forming unit quantification using the double-layer LB plaque assay. The phage burst size was calculated from the plotted curve following the observed latent period.

Thermal stability was assessed by incubating the phage at 30°C, 40°C, 50°C, 60°C, 70°C, and 80°C for 1 h, followed by titer determination. For pH stability testing, phage suspensions were incubated in buffers spanning pH 2–13 for 1 h, and aliquots at each pH were analyzed for phage titer. Chloroform susceptibility, a measure of structural stability, was evaluated by mixing 1% (vol/vol) phage lysate with chloroform, incubating at room temperature for 30 min, and determining residual phage titer after thorough mixing.

### Preparation of phage vB_SalP_SE29 microcapsules

#### Single-factor experiment

The method was slightly modified from the previous research (18), we prepared microencapsulated phage microspheres. In this process, sodium alginate (Solarbio, China) and gelatin (Solarbio, China) were each dissolved in a 50 mmol/L Tris-HCl (Solarbio, China) solution and sterile water, respectively. The solutions were heated to 60°C and stirred until completely dissolved. Subsequently, the phage was added and mixed thoroughly until a uniform solution was achieved. This mixture was then dripped into a calcium chloride (Macklin, China) solution using a syringe, allowed to gel completely, and filtered to isolate the microspheres. To assess the influence of sodium alginate concentration, gelatin concentration, and calcium chloride concentration on the encapsulation efficiency of the phage, the encapsulation rate was established as the evaluation criterion. Under controlled conditions, optimal parameters for phage microencapsulation were determined through a univariate analysis.

#### Orthogonal optimization test

Orthogonal optimization experiments were conducted to analyze the effects of three factors—sodium alginate concentration, gelatin concentration, and calcium chloride concentration—on the encapsulation rate of microencapsulated phage vB_SalP_SE29 microspheres. The levels of these influencing factors were presented in Table 1 for reference (19). These experiments aimed to identify the optimal conditions for encapsulating the phage vB_SalP_SE29, thereby achieving the best preparation process parameters.

**TABLE 1:** Orthogonal optimization assay for microencapsulated phage preparation

Experimental group	A sodium alginate (g/L)	B gelatin (g/L)	C CaCl_2_ (g/L)	Y encapsulation efficiency (%)
1	0.1	0.7	0.165	72.82
2	0.1	0.8	0.22	75.24
3	0.1	0.9	0.165	65.21
4	0.2	0.7	0.22	72.4
5	0.2	0.7	0.11	75.84
6	0.2	0.8	0.165	86.61
7	0.2	0.9	0.11	70.23
8	0.2	0.9	0.22	71.04
9	0.3	0.7	0.165	75.7
10	0.3	0.8	0.11	80.4
11	0.3	0.9	0.165	72.58
12	0.2	0.8	0.165	85.42
13	0.2	0.8	0.165	85.32
14	0.2	0.8	0.165	86.52
15	0.2	0.8	0.165	84.62

### Encapsulation rate of microencapsulated phages

Wet or dried phage microspheres were placed in the microencapsulated phage lysis solution (SM, NaCl 5.8 g; MgSO_4_·7H_2_O [Macklin, China] 2 g; Tris·HCl 1 mol/L [Solarbio, China] pH 7.5, 50 mL; gelatin 0.1 g; sterile water to a final volume of 1 L). The solution was completely dissolved at room temperature and then filtered through a 0.22 μm membrane (Millipore, USA). The microencapsulated phage lysis solution containing phages was continuously diluted, and phages were cultured using the double-layer LB agar plate method to produce plaques. The phage titer was determined by counting the plaques (20).

The formula for calculating titer was as follows: potency (PFU/mL) = number of plaques × dilution factor × 10.

Additionally, the encapsulation rate of phages in the microencapsulated phage lysis solution was calculated using the following formula: encapsulation rate (%) = (amount of released phages from microspheres/input amount of phages during microencapsulation preparation) × 100%.

### Morphology of microencapsulated phages

Observation of microencapsulated phage vB_SalP_SE29 microparticles was performed using an optical microscope (Olympus), followed by morphological examination using scanning electron microscopy (Thermofisher Apreo S Lovac). Transmission electron microscopy (TEM; HT-7800) was used to examine the microstructure of phage vB_SalP_SE29. To determine whether the phages were successfully encapsulated inside the microencapsulated phage pellets, we sectioned the pellets and observed them using a TEM. First, the microencapsulated phage pellets were fixed in PBS buffer containing 4% (vol/vol) glutaraldehyde and 0.05% (wt/vol) ruthenium red for 4 h. After fixation, the pellets were rinsed with PBS buffer. Subsequently, a secondary fixation was performed in the same PBS buffer supplemented with ruthenium red using 2% (wt/vol) osmium tetroxide for 4 h. Finally, after rinsing with PBS buffer, the pellets were dehydrated through a graded ethanol series (25%–100%, [vol/vol]) and embedded in LR White resin. Ultrathin sections (60 nm) were cut using an ultramicrotome (Leica UC7, Germany), then stained with uranyl acetate and lead citrate. Electron micrographs were captured using an FEI Tecnai Spirit TEM (FEI, the Netherlands) operated at 100 kV under standard operating conditions (21). To further characterize the microencapsulated phages, compositional analysis was conducted using an infrared spectrometer (Bruker Alpha II).

### Stability of phage vB_SalP_SE29 microencapsulated microspheres

#### pH stability

Microspheres containing microencapsulated phage vB_SalP_SE29 (1 g) were placed in LB (9 mL) liquid medium at various pH levels (pH 2 to pH 12), which had been preheated to 37°C. The mixture was then incubated at 37°C with continuous shaking at 120 rpm for 1 h. After the addition of the microencapsulated phage lysate, complete lysis of the solution occurred. The lysate was subsequently filtered through a 0.22 μm membrane, and the titer of the microencapsulated phage vB_SalP_SE29 was determined using the double-layer agar plate method. This experiment was conducted three times, and the average titer was calculated.

#### Simulated gastric fluid stability

Microspheres containing microencapsulated phage vB_SalP_SE29 (1 g) were placed in 9 mL of simulated gastric fluid (SGF) at pH 2 (SGF, comprises 2 g NaCl; 3.2 g pepsin [Merck, Germany]; sterile water to a final volume of 1 L) and incubated at 37°C with shaking at 120 rpm for 5, 15, 30, 60, and 90 min. Following incubation, the microencapsulated phage was lysed and filtered through a 0.22 μm membrane filter. The titer of the phage vB_SalP_SE29 was determined using a double-layer agar plate assay. The experiment was conducted three times, and the average titer was calculated.

#### Simulated BS stability

Microspheres containing microencapsulated phage vB_SalP_SE29 (1 g) were placed in 9 mL of 1% or 2% (wt/vol) bile salts (BS, porcine bile extract [Sigma-Aldrich, USA] 1 or 2 g; sterile water 100 mL). The microspheres were then incubated at 37°C with shaking at 120 rpm for 1 h and 3 h, respectively. Following incubation, the microencapsulated phage was lysed and filtered through a 0.22 μm membrane filter. The titer of the phage vB_SalP_SE29 was determined using a double-layer agar plate assay. The experiment was conducted three times, and the average titer was calculated.

#### Simulated intestinal fluid release rate

Microspheres containing microencapsulated phage vB_SalP_SE29 (1 g) were placed in 9 mL of simulated intestinal fluid (SIF, KH_2_PO_4_ [Macklin, China] 6.8 g; trypsin [Solarbio, China] 10 g; sterile water to make 1 L; pH 6.8). The mixture was then incubated at 37°C with shaking at 120 rpm for 12 h. Following incubation, the microencapsulated phage was lysed and filtered through a 0.22 μm membrane filter. The titer of the phage vB_SalP_SE29 was determined using a double-layer agar plate assay. The experiment was conducted three times, and the average titer was calculated.

#### Phage storage stability

The efficacy of vB_SalP_SE29 from three different production batches was evaluated by storing the microcapsules at 4°C for 6 weeks. Portions of the microcapsules were sampled every 7 days and tested in a phage lysis buffer. The experiment was conducted three times, and the average efficacy was calculated.

### Treatment of the phage vB_SalP_SE29 *in vivo*

To determine the minimum lethal dose (MLD), 10 rats from each group were injected with *Salmonella* S.E-29 (at concentrations of 1 × 10⁵, 1 × 10⁶, 1 × 10⁷, 1 × 10⁸, and 1 × 10⁹ CFU/mL), while the control group was not inoculated with bacteria. The 2× MLD was used as the infective inoculum.

Forty female SD rats were divided into four groups of 10 rats each (PBS group, control group, microencapsulated phage treatment group, and free phage treatment group). The rats were intraperitoneally injected with *Salmonella* S.E-29 at an inoculum of 2× MLD (2 × 10⁹ CFU/mL) to cause infection. After 8 h of infection, the rats were treated with 1 mL of PBS sterile buffer (oral gavage), 1 mL of free phage vB_SalP_SE29 (1 × 10⁷ PFU/rat) administered by oral gavage, or 1 g of microencapsulated phage vB_SalP_SE29 (1 × 10⁷ PFU/rat) also administered by oral gavage. Additionally, a separate positive control group received free phage vB_SalP_SE29 (1 × 10⁷ PFU/rat) via intraperitoneal injection. The rats were closely observed for 7 days following treatment.

The expression levels of TNF-α, IL-1β, and IL-10 in the liver, spleen, and intestinal tissues of rats in different groups were quantified using quantitative polymerase chain reaction (qPCR). The specific information of the primers is shown in Table 2.

**TABLE 2:** RT-qPCR primer sequences

Gene	qRT-PCR primer sequences
gaph	P1:GCCTTCCGTGTTCCTACCC
	P2:TGCCTGCTTCACCACCTTC
IL-1β	P1:TCAGGCAGGCAGTATCACTCATTG
	P2:AAGAAGGTGCTCATGTCCTCATCC
IL-10	P1:GGTTGCCAAGCCTTATCGGAAATG
	P2:GCCGCATCCTGAGGGTCTTC
TNF-α	P1:ACGCTCTTCTGTCTACTGAACTTCG
	P2:TGGTTTGTGAGTGTGAGGGTCTG

The duodenum, jejunum, and ileum tissues of rats in each group were randomly collected at 7 days post-treatment (all rats in the control group had died within 3 days), and histopathological analysis was performed.

### Statistical analysis

The statistical data in this study were analyzed using one-way analysis of variance (ANOVA), Student’s t-test, and simple survival analysis (Kaplan-Meier). All images were generated with GraphPad Prism 8.0 (GraphPad Software, USA). The error bars represent the standard deviation of the mean. *P < 0.05 indicates a significant difference in the data; **P < 0.01, ***P < 0.001, and ****P < 0.0001 indicate a very significant difference in the data.

## RESULTS

### Biological characteristics of vB_SalP_SE29 phage

**Fig 1:**
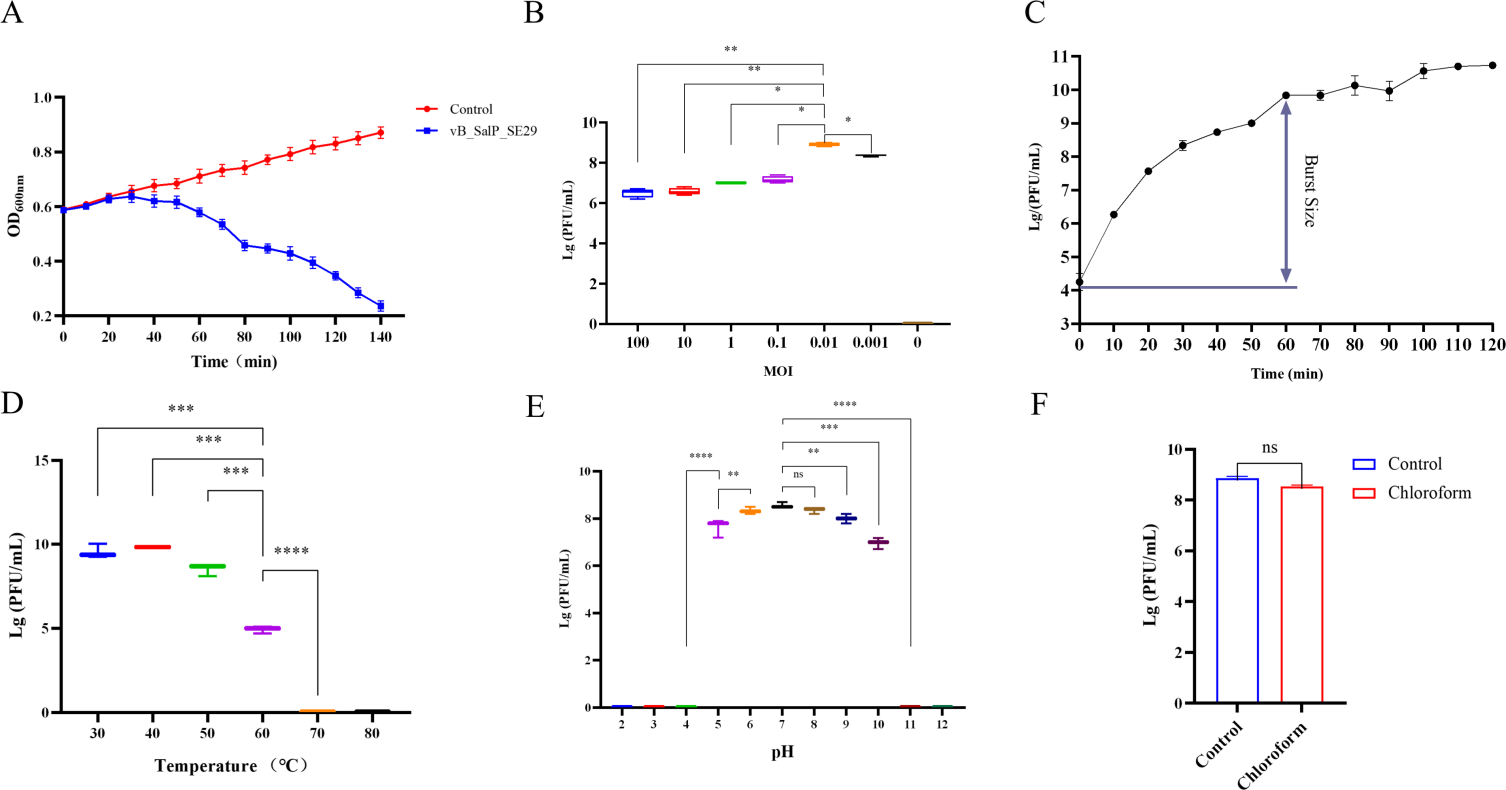
Biological characterization of phage vB_SalP_SE29. (**A**) *In vitro* lytic activity. The lytic ability of the phage against host bacterium SE29 was determined over 140 min. (**B**) Optimal MOI. Phage/host bacterium ratios were tested to determine the optimal MOI. (**C**) One-step growth curve. Phage titer changes were monitored over a 2 h period. (**D**) Thermal stability. Phage stability was evaluated at different temperatures. (**E**) pH stability. Phage survival was analyzed in environments with varying pH values. (**F**) Chloroform resistance. Phage tolerance to chloroform was measured. Data are presented as mean ± standard deviation (*n* = 3). Each assay was repeated three times, and statistical analysis was performed to compare microencapsulated and free phage groups. ns = no significant difference; *P < 0.05; **P < 0.01; ***P < 0.001; ****P < 0.0001*.*

*In vitro* lysis results showed that vB_SalP_SE29 exhibited strong lytic activity against the host bacterium *Salmonella* S.E-29 (Fig. 1A). The optimal MOI was determined to be 0.01 (Fig. 1B). The one-step growth curve indicated that the phage had a latent period of approximately 30 min, a rise period of about 40 min, and a complete lysis cycle lasting roughly 70 min (Fig. 1C).

Thermal stability analysis revealed that the phage remained active between 30°C and 50°C (Fig. 1D), with reduced activity at 60°C and complete inactivation at 70°C–80°C. pH stability testing showed that vB_SalP_SE29 was stable within the pH range of 5–10, exhibiting optimal activity at pH 6–8 (Fig. 1E). Additionally, the phage’s insensitivity to chloroform further confirmed the absence of a lipid membrane structure (Fig. 1F).

### Optimal parameters for phage microencapsulation

**Fig 2:**
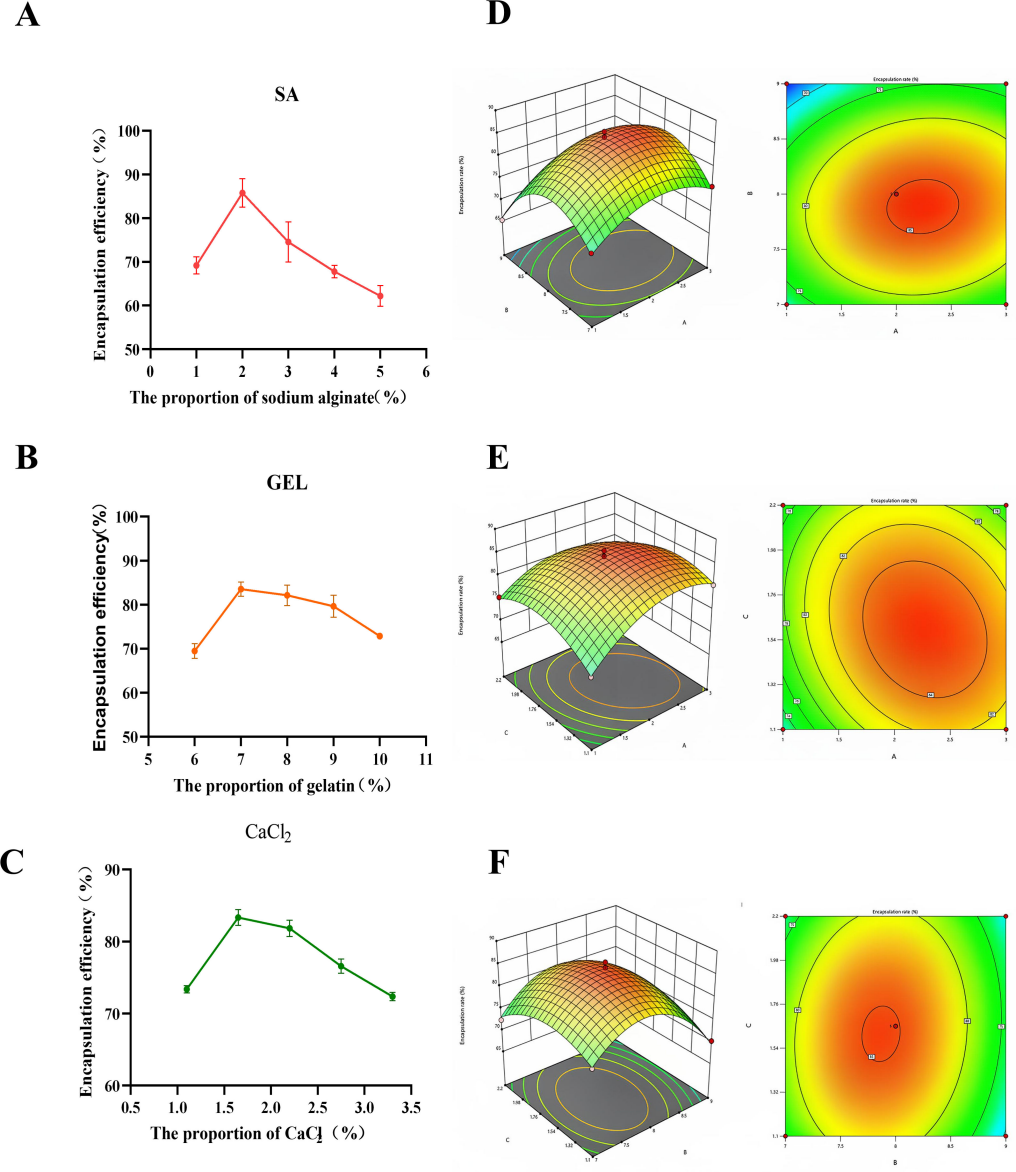
The optimum concentrations of sodium alginate, gelatin, and calcium chloride were selected by one-way as well as orthogonal test analyses. (**A**) Effect of sodium alginate concentration on encapsulation rate. (**B**) Effect of gelatin concentration on encapsulation rate. (**C**) Effect of CaCl_2_ concentration on encapsulation rate. (**D**) Response surface and contour plots of the effects of sodium alginate concentration and gelatin concentration on the encapsulation rate of microencapsulated phages. (**E**) Response surface and contour plots of the effects of sodium alginate concentration and CaCl_2_ concentration on the encapsulation rate of microencapsulated phages. (**F**) Response surface and contour plots of the effects of gelatin concentration and CaCl_2_ concentration on the encapsulation rate of microencapsulated phages.

We determined the most suitable concentrations of each substance through individual factor experiments. The results (Fig. 2A through C) demonstrated that the highest encapsulation efficiency, influenced by a single factor, was attained with sodium alginate at a concentration of 2%, gelatin at 7%, and calcium chloride at 1.65%. To further enhance the encapsulation efficiency, we conducted orthogonal optimization. The outcomes (Fig. 2D through F) indicated that the optimal concentrations for the preparation of microencapsulation phage vB_SalP_SE29 microspheres were sodium alginate at 2%, gelatin at 8%, and calcium chloride at 1.65%, resulting in a maximum encapsulation efficiency of 86.52%.

### Microstructure of microencapsulation phages

**Fig 3:**
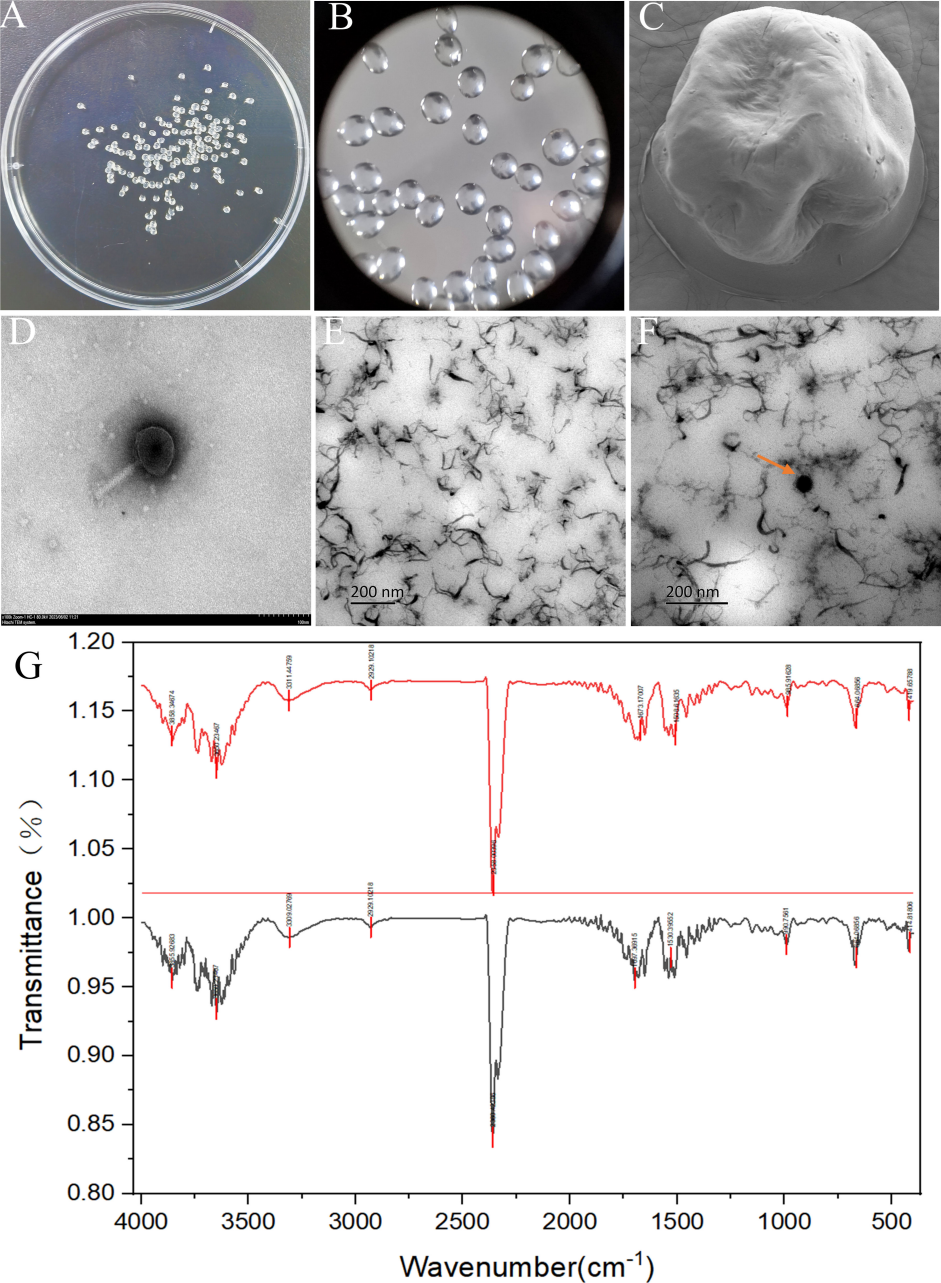
Images of microencapsulated phage vB_SalP_SE29. (**A**) Physical image of microencapsulated phage. (**B**) Optical micrograph of microencapsulated phage. (**C**) Scanning electron micrograph of microencapsulated phage. (**D**) TEM image of the phage (magnification: 100.0K×; scale bar: 100 nm). (**E**) Transmission electron micrograph of a section of non-phage-encapsulated microencapsulated spheres (scale bar: 200 nm). (**F**) Transmission electron micrograph of a section of phage-encapsulated microencapsulated spheres (scale bar: 200 nm). (**G**) Infrared spectrum of microencapsulated phage.

The microstructure of microencapsulated phage vB_SalP_SE29 microspheres was depicted in Fig. 3A through C. These microspheres exhibited a uniform size, near-spherical shape, and a smooth, slightly concave surface, with an average particle size of 1.27 mm. Figure 3D shows micrographs of phage vB_SalP_SE29 imaged by TEM. As shown in Fig. 3F, TEM provided physical evidence that the microencapsulated phage spheres contained phages. The infrared spectrum of microencapsulated phage vB_SalP_SE29 microspheres was shown in Fig. 3G, where the stretching vibration of the O-H bond of alginate appeared in the range of 3,250–3,750 cm^−1^, and the vibrations observed in the range of 1,600–1,500 cm^−1^ were attributed to the asymmetric and symmetric stretching vibrations of carboxylate ions (22). The absorptions at 1,900–1,650 cm^−1^ were related to the stretching of carbon-oxygen bonds in aldehydes, ketones, and acids, and the peaks at 1,600–1,430 cm^−1^ were associated with the C-C benzene ring stretching of aromatic groups (23). The identified functional groups were consistent with the components of sodium alginate, gelatin, and calcium chloride.

### Biological stability of microencapsulated phages

**Fig 4:**
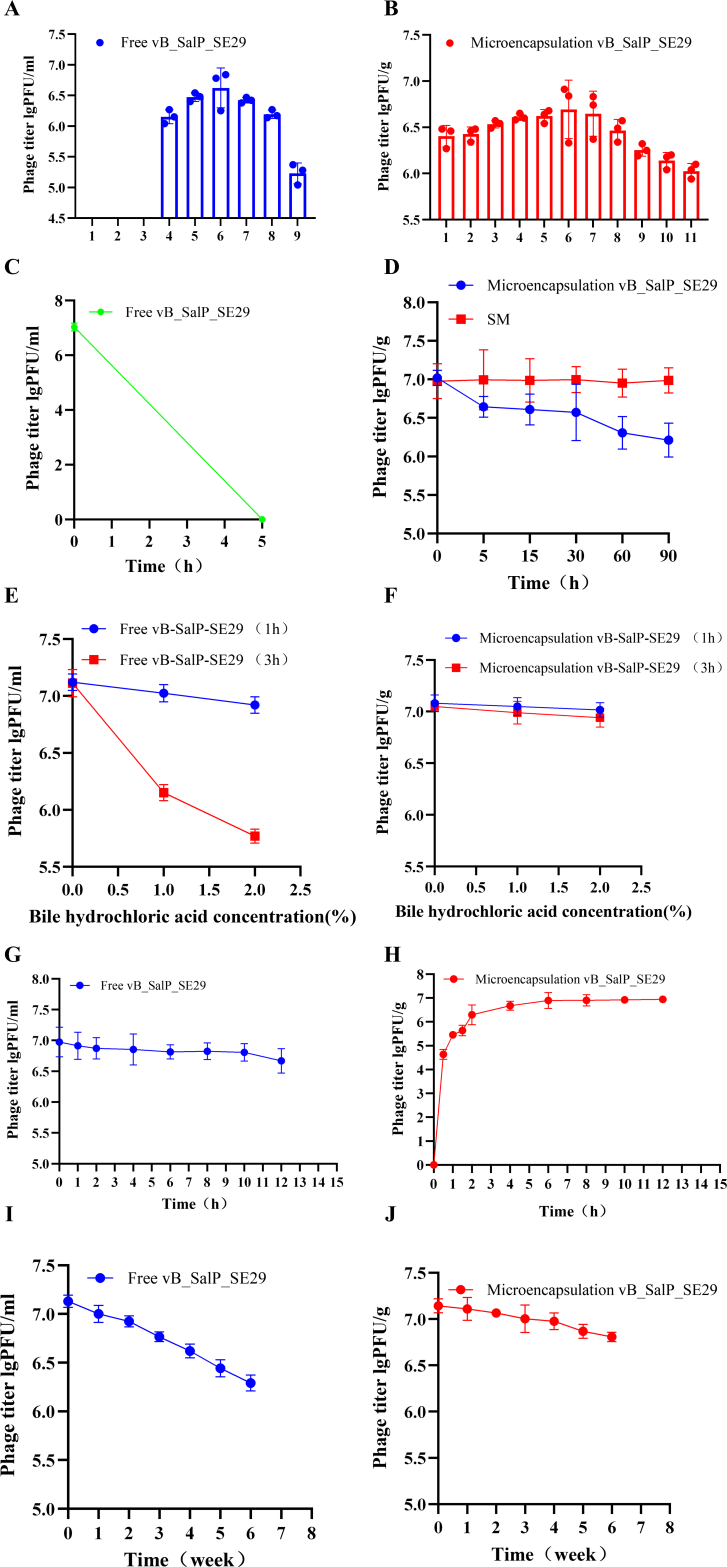
Biological characteristics of microencapsulated phage vB_SalP_SE29. (**A**) pH stability of free phages. (**B**) pH stability of microencapsulated phages. (**C**) Stability of free phages in SGF. (**D**) Stability of microencapsulated phages in SGF; SM: The titer of phages detected after the microencapsulated phages are lysed in SM buffer. (**E**) Stability of free phages in simulated BS. (**F**) Stability of microencapsulated phages in simulated BS. (**G**) Release characteristics of free phages in SIF. (**H**) Release characteristics of microencapsulated phages in SIF. (**I**) Stability of free phages during storage. (**J**) Stability of microencapsulated phages during storage. Each value in the figure represents the mean ± standard deviation (*n* = 3). After incubation in different pH, SGF, BS, or SIF, the stability of microencapsulated and free phages in different pH, SGF, or BS and the release rate in SIF were tested by calculating the titer (potency) of phages at different pH or time points. Microencapsulated and free phages were stored at 4°C, and the titer (potency) was measured weekly. Each determination was performed in triplicate. Statistical analysis was used to compare the microencapsulated phage group and the free phage group. ns, no significant difference; *P < 0.05; **P < 0.01; ***P < 0.001*.*

As depicted in Fig. 4A and B, free phages exhibited relative stability within a pH range of 5–10 but were rapidly inactivated when the pH fell below 5 or rose above 10. The efficacy of microencapsulated phages remained unchanged across the pH range of 3–9, suggesting that microencapsulation enhanced phage stability in varying pH conditions. Figure 4C and D illustrated the vulnerability of free phages in strongly acidic environments, consistent with the outcomes of the pH stability tests. Conversely, the stability of microencapsulated phages in SGF at pH 2 was markedly improved; after 90 min, the titer decreased by only 0.79 lg PFU/g, indicating the relative stability of microencapsulated phages in this environment. Figure 4E and F demonstrated that, after 1 h of incubation, there was no significant decrease in the titer of free phages at 1% and 2% BS. However, after 3 h of incubation, a substantial decline in titer was observed. In contrast, microencapsulated phages showed no significant reduction in titer after either 1 or 3 h of incubation at 1% and 2% BS concentrations, indicating their superior stability compared to free phages. Figure 4G and H show that no significant decrease in titer occurred after 12 h of incubation of free phages in SIF, confirming the high stability of free phages in SIF, which was essential for subsequent release experiments. In contrast, microencapsulated phages demonstrated favorable release characteristics in SIF, with phage released reaching 6.94 lg PFU/mL after 12 h of incubation and a total release rate of 93.325%, approaching complete release. The results in Fig. 4I and J indicated that, after 6 weeks of storage at 4°C, the titer of free phages decreased by 0.591 lg PFU/mL, while the titer of microencapsulated phages was reduced by only 0.146 lg PFU/g. These findings suggested that encapsulation significantly enhanced phage stability, resulting in prolonged storage longevity.

### Microencapsulated phages showed efficacy in the treatment of *Salmonella* infections *in vivo*

**Fig 5:**
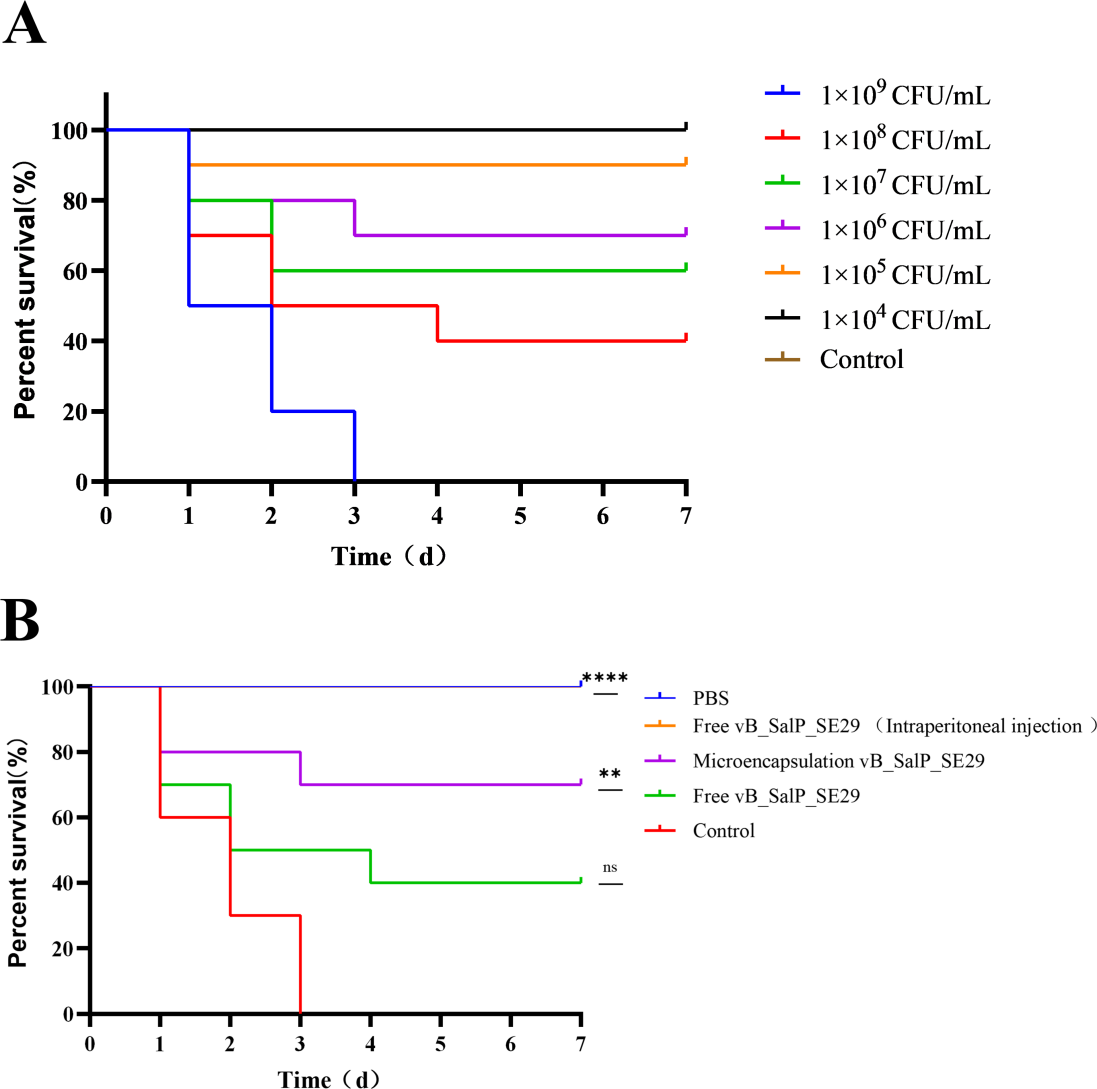
Microencapsulated phage vB_SalP_SE29 is effective in the treatment of rats infected with *Salmonella* S.E-29-induced enteritis. (**A**) Determination of the MLD of *Salmonella* S.E-29 in rats. The 2× MLD (2 × 10⁹ CFU/mL) was determined by intraperitoneal injection with a gradient of bacterial concentrations (1 × 10⁴–1 × 10⁹ CFU/mL). Since the survival rates of both the group with a concentration of 10⁴ CFU/mL and the control group were 100%, the two survival rate curves overlapped. (**B**) Survival rate of rats treated with microencapsulated phage vB_SalP_SE29 and free phage vB_SalP_SE29. *Salmonella* S.E-29 was administered via oral gavage. In the treatment groups, free vB_SalP_SE29 was administered via intraperitoneal injection and oral gavage, while microencapsulated vB_SalP_SE29 was administered via oral gavage. Since the survival rates of both the free phage (intraperitoneal injection) group and the PBS group were 100%, the two survival rate curves overlapped. Statistical analysis was used to compare the control group, the microencapsulated phage group, and the free phage group. ns, no significant difference; *P < 0.05; **P < 0.01; ***P < 0.001; ****P < 0.0001*.*

**Fig 6:**
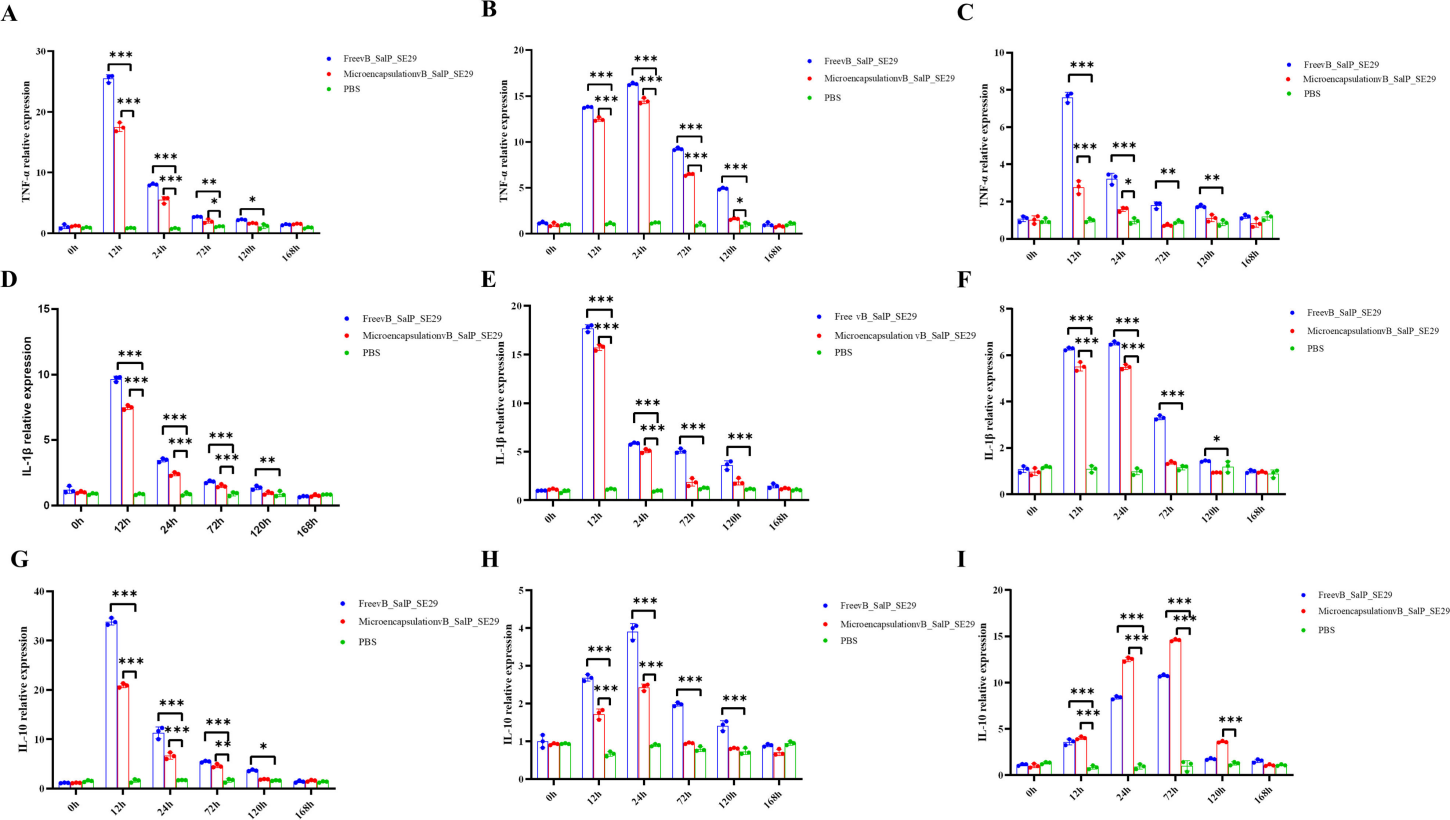
Transcriptional changes of specific genes in rat liver, spleen, and intestine after treatment with microencapsulated phage vB_SalP_SE29. (**A**) TNF-α in liver tissue. (**B**) TNF-α in spleen tissue. (**C**) TNF-α in intestinal tissue. (**D**) IL-1β in liver tissue. (**E**) IL-1β in spleen tissue. (**F**) IL-1β in intestinal tissue. (**G**) IL-10 in liver tissue. (**H**) IL-10 in spleen tissue. (**I**) IL-10 in intestinal tissue. Changes in inflammatory factors in various tissues of rats within 168 h after administration of *Salmonella* to rats. Each value in the figure represents mean ± SD (*n* = 3). Statistical analysis was used to compare the microencapsulated phage group and the free phage group. ns, no significant difference; *P < 0.05; **P < 0.01; ***P < 0.001*.*

The results indicated that the intraperitoneal injection of 1.0 × 10⁷ PFU/Rat of phage vB_SalP_SE29 achieved a survival rate of 100% in rats infected with 2× MLD *Salmonella* S.E-29 (Fig. 5A). Additionally, treatment with microencapsulated phage vB_SalP_SE29 significantly improved the survival of rats with *Salmonella* infection, with a survival rate of 70% in the microencapsulated phage group compared to 40% in the free phage group (Fig. 5B). qPCR was used to measure the expression levels of IL-1β, IL-10, and TNF-α in the liver, spleen, and intestines of rats. As shown in Fig. 6, the pro-inflammatory cytokine TNF-α in the liver, spleen, and intestines in the microencapsulated phage group reached levels comparable to those of the PBS group at 120 h, while the free phage group reached similar levels to the PBS group at 168 h (Fig. 6A through C). The expression levels of IL-1β in liver, spleen, and intestinal inflammatory cells in both the microencapsulated phage and free phage groups were significantly elevated at 24 h, peaking at this time; however, throughout the 168 h observation period, the overall levels in the microencapsulated phage group remained lower than in the free phage group (Fig. 6D through F). Except in the liver, the levels of the anti-inflammatory and immunosuppressive factor IL-10 in the spleen and intestine peaked at 24 h (Fig. 6G through I).

**Fig 7:**
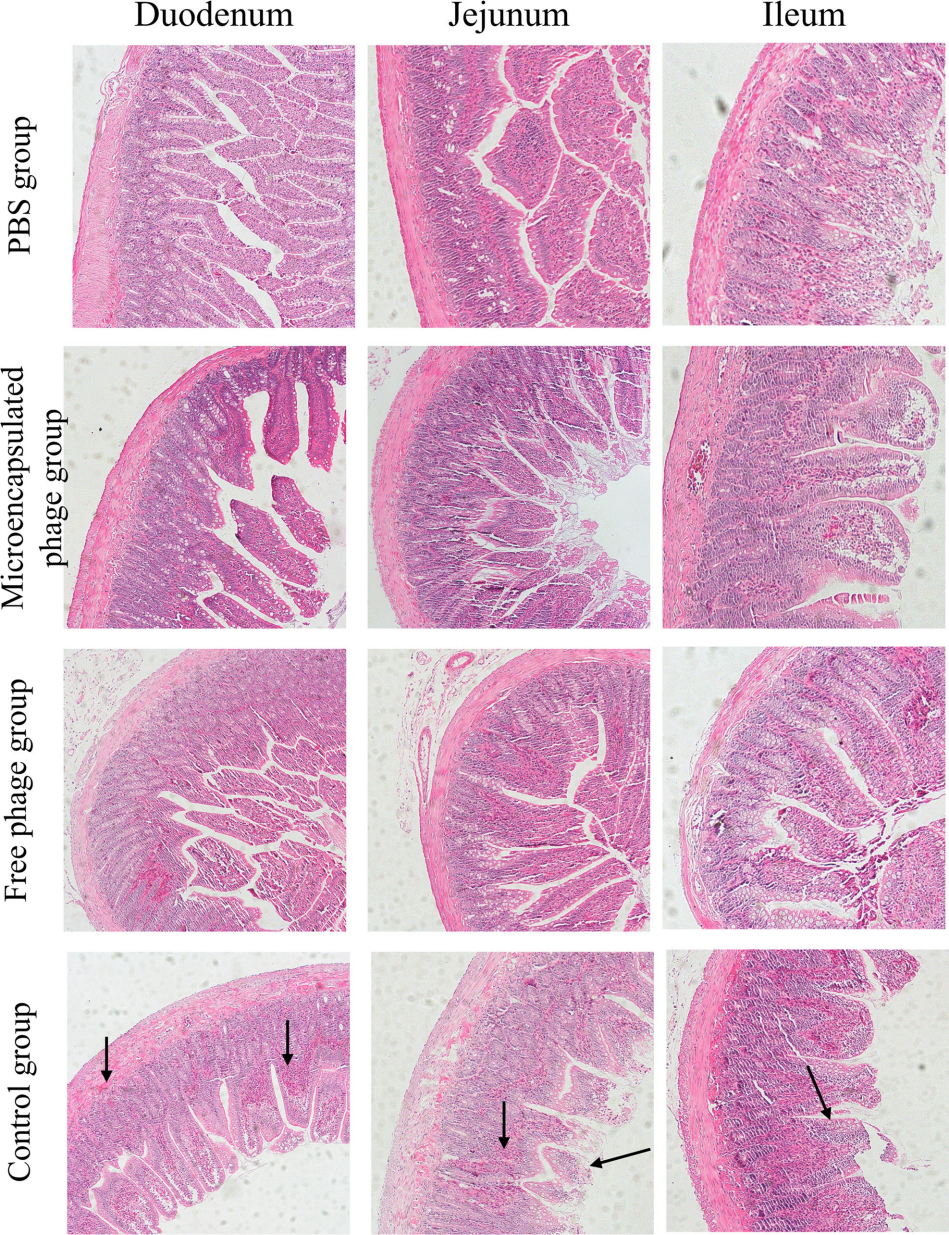
H&E staining (100×) of pathological sections of duodenum, jejunum, and ileum tissues of rats in each group.

Histological changes in the duodenum, jejunum, and ileum of rats in each treatment group were assessed using H&E staining (Fig. 7). In the control group, hemorrhagic changes were observed in the duodenal lamina propria and submucosa, with partial rupture, loss, and inflammatory cell lesions in the jejunum, and hemorrhage at the tips of the intestinal villi. The ileum showed only partial shedding of epithelial cells. Compared to the control group, the free phage group showed improvements in intestinal hemorrhaging, reduced inflammatory cells, and more orderly villi arrangement. Compared to both the control and free phage groups, the microencapsulated phage group showed further improvements in the symptoms mentioned above. Overall, these results suggested that microencapsulated phage vB_SalP_SE29 alleviated the pathological damage caused by *Salmonella* S.E-29 infection in the rat intestine.

## DISCUSSION

The misuse of antibiotics has led to the emergence of drug-resistant bacteria, thereby rekindling interest in phage therapy. Phage therapy could be administered via intraperitoneal injection, aerosol spray, oral consumption, and other methods (24–27). Although oral administration presented advantages in terms of convenience and storage, it also posed significant challenges. Several studies indicated that the principal barrier to phage entry into the body was the highly acidic gastric environment, which resulted in phage inactivation upon exposure to gastric juice, thereby restricting the broader application of oral therapy (28–30). To ensure that phages retained their high bactericidal activity after entering the body, microencapsulation technology presented a viable solution for encapsulating phages.

In this investigation, we selected sodium alginate, gelatin, and calcium chloride as the materials for microencapsulation. Our experiments revealed that the optimal concentrations for preparing microspheres encapsulating the phage vB_SalP_SE29 were 2% sodium alginate, 8% gelatin, and 1.65% calcium chloride. This formulation resulted in the highest encapsulation efficiency, reaching 86.52%. Sodium alginate, a biopolymer known for its excellent biocompatibility, affordability, and low toxicity, could gelatinize with Ca^2+^ ions in water at room temperature under gentle conditions. Its pH sensitivity allowed it to contract in acidic environments and dissolve in alkaline conditions, thereby offering protection to the phages. The concentration of sodium alginate played a pivotal role in the preparation of microencapsulated phage microspheres, influencing the mechanical strength, shape, encapsulation efficiency, and ease of formation of the microspheres (31–33). Gelatin, known for its significant gelling ability, was often added to polysaccharide solutions to obtain composite gels with different functionalities. CaCl_2_ ions were commonly used cross-linkers and are essential in forming a gel network with sufficient sodium alginate under mild conditions for microcapsule formation (34). These three materials were inexpensive, easy to prepare, and suitable for large-scale production without the need for strict preparation environments. O’Connell employed alginate encapsulation to treat and prevent enterohemorrhagic *Escherichia coli* infections, such as *E. coli* O157 (35). Ahmadi used sodium alginate, arabic gum, and gelatin to prepare phage microencapsulation to maximize their vitality during thermal processing (36). In addition, there were studies indicating that other chemical materials were used to encapsulate phages. Samananda could efficiently deliver phage drugs by encapsulating phages with nanoemulsions (37). Nanoemulsions had the characteristic of better compatibility. Zhou used alginate (ALG)/κ-carrageenan (CG) to prepare microencapsulated phages, which could enhance the antibacterial effect of phages (28). Compared with existing studies (e.g., liposome or nanoemulsion systems), this protocol achieved a high encapsulation efficiency of 86.52% through orthogonal optimization, surpassing the reported 70%–80% in the literature. Additionally, stability in simulated gastric fluid (pH 2) was significantly improved, with only a 0.79 lg PFU/g titer decrease, which is superior to the 1.5 lg PFU/g decline reported by Colom et al. Moreover, this material is cost-effective and suitable for large-scale production. So far, a variety of phage coating materials have been well applied. In the future, we would consider using more materials to coat phages to enhance the therapeutic effect of phages.

Based on our previous findings, we had successfully obtained phages with a high encapsulation rate. However, a crucial aspect of achieving therapeutic effectiveness through oral phage administration was their ability to remain active in gastric acid. To address this concern, we conducted experiments to evaluate the relevant biological characteristics of these phages. It was well known that free phages were sensitive to variations in pH, particularly vulnerable to low pH conditions, with most being inactivated when the pH fell below 3.5 (4). In contrast, the microencapsulated phage vB_SalP_SE29 developed in this study exhibited remarkable resistance to both acidic and alkaline environments within the pH range of 4–9. When compared to free phages, the microencapsulated form demonstrated greater stability in simulated gastric fluid and in the presence of bile salts, while also maintaining a high release rate in simulated intestinal fluid. Furthermore, after being stored at 4°C for 6 weeks, the microencapsulated phages showed a marked improvement in stability compared to their free counterparts. The microencapsulation process had effectively shielded the phages from external interference, preserving their activity and extending their shelf life, thereby enhancing their suitability for practical applications. Research conducted by Ferreira on the use of microencapsulated phages for treating enterotoxigenic *E. coli* demonstrated good stability and a high release rate in simulated gastric and intestinal fluids in comparison to free phages (38). Similarly, Lorenzo-Rebenaque compared microencapsulated and non-encapsulated phages, examining their retention in the chicken gastrointestinal tract (39). The findings indicated that microencapsulated phages provided significant protection, potentially facilitating the delivery of high doses to the cecum. These studies collectively suggested that microencapsulation significantly enhances the survival of phages in the gastrointestinal tract compared to their free forms, corroborating the results of our current experiments.

The microencapsulated phage demonstrated promising biological characteristics *in vitro*; however, its efficacy in treating related diseases remains a critical issue that warrants further investigation. An experiment examining the effect of microencapsulated phages on rat survival rates revealed a 30% increase in the survival rate of the microencapsulated phage group compared to the free phage group. This finding suggested that the microencapsulation of phages enhances survival outcomes in rats. In this study, we analyzed the relative expression levels of IL-1β, IL-10, and TNF-α in the liver, spleen, and intestines of the rats. TNF-α, a pro-inflammatory cytokine, plays a key role in normal inflammatory and immune responses. IL-1β was an inflammatory cytokine involved in various physiological processes, including those of the hematopoietic, nervous, and endocrine systems, as well as in anti-tumor responses (40). Conversely, IL-10 acts as an anti-inflammatory and immunosuppressive factor that regulates cell growth and differentiation and contributes to inflammatory and immune responses. Notably, the levels of these cytokines in the microencapsulated phage treatment group peaked at either 12 or 24 h post-treatment before declining more rapidly, ultimately aligning with the levels observed in the PBS control group. This pattern indicated that microencapsulated phages were more effective than their free counterparts in reducing inflammatory markers and expediting recovery in rats. In the later stages of phage treatment, the body’s immune system recognized the phages as foreign antigens, triggering a self-regulatory response. A study by Xue investigating the treatment of pathogen-infected mice with phages found that levels of various inflammatory factors returned to normal, significantly lower than those in the control group (41). Similarly, research by Yin on the treatment of *E. coli*-infected rats using microencapsulated phages indicated an earlier and more pronounced decrease in cytokine levels compared to the free phage group (21). Collectively, these studies underscore the enhanced anti-inflammatory efficacy of microencapsulated phages.

### Conclusion

**Fig 8:**
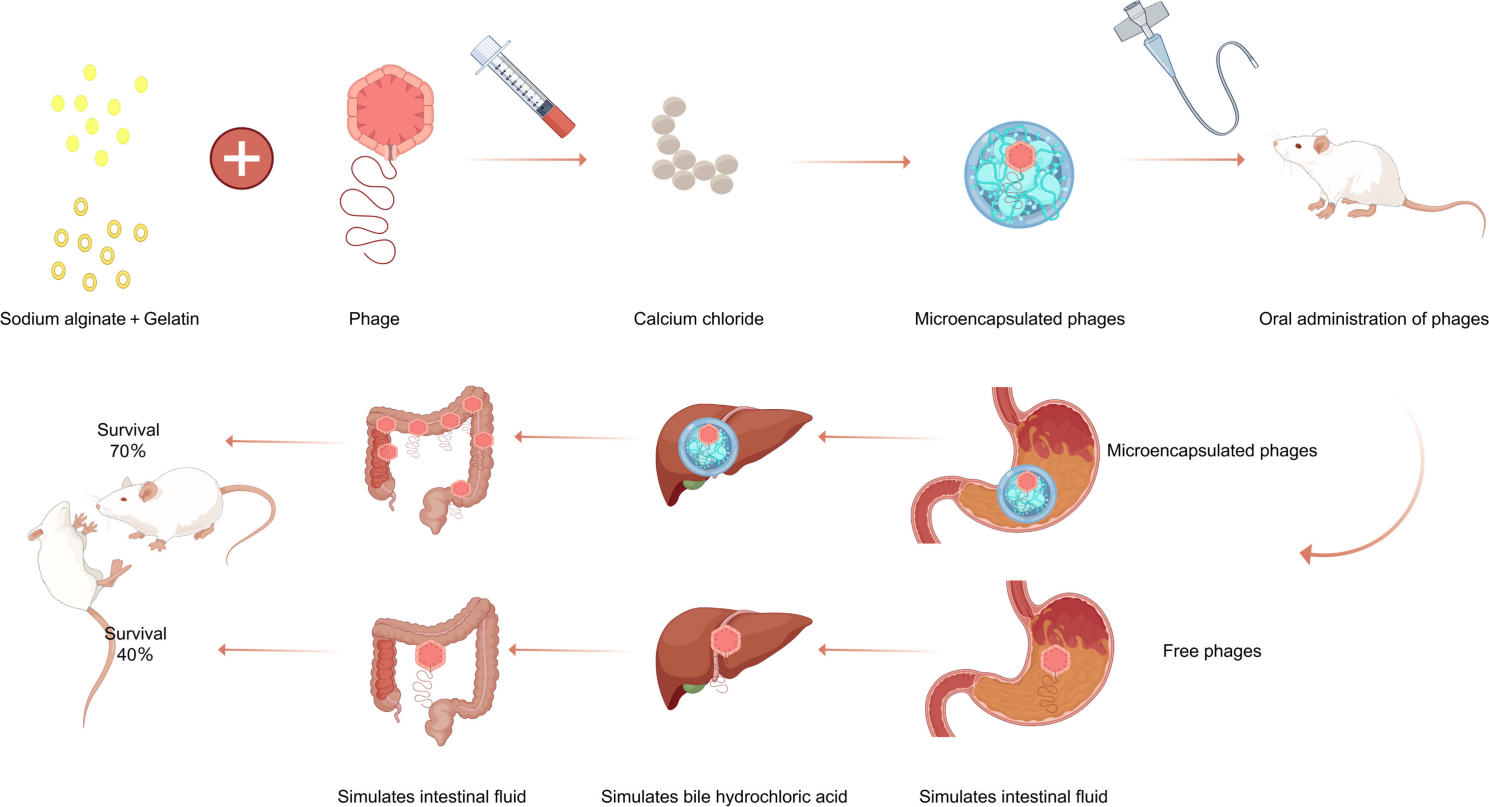
Experimental flow chart. This figure was drawn using Figdraw.

In summary, the microencapsulated phage vB_SalP_SE29, developed in this study using a formulation of 2% sodium alginate, 8% gelatin, and 1.65% calcium chloride, achieved an encapsulation efficiency of 86.52%. Furthermore, the microencapsulated phage exhibited significant tolerance to both acidic and alkaline environments, demonstrated stability in simulated gastric fluid and bile hydrochloric acid, exhibited favorable release characteristics in simulated intestinal fluid, and maintained stability during preservation. Additionally, this microencapsulated phage effectively treated *Salmonella* infections in rats. Consequently, the vB_SalP_SE29 microspheres demonstrated considerable potential for the development and application of this phage as a safe and effective bio-antimicrobial agent.The technical route is shown in Fig. 8.
