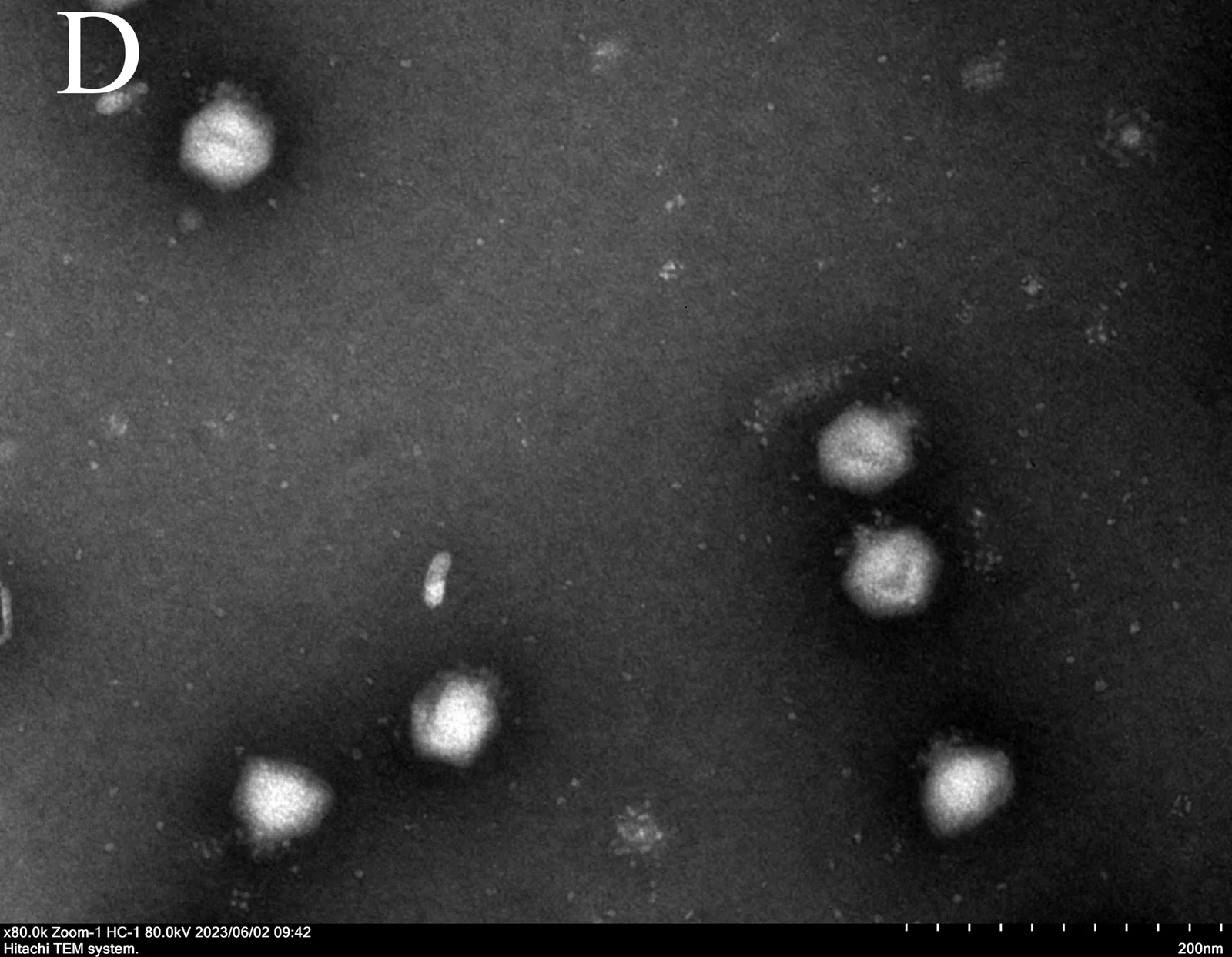# Correction for Cheng et al., “Preparation of sodium alginate-coated microencapsulated phage vB_SalP_SE29 and evaluation of its therapeutic effect on *Salmonella* enteritidis infection”

**DOI:** 10.1128/aem.01329-26

**Published:** 2026-08-06

**Authors:** Yan Cheng, Jiahao Yu, Xiaochen Ren, Ruiqi Liang, Bingmei Du, Jinhong Li, Sayed Haidar Abbas Raza, Wuwen Sun, Dongxing Zhang, Lei Zhang

## AUTHOR CORRECTION

Volume 92, no. 3, e02263-25, 2026, https://doi.org/10.1128/aem.02263-25. Figure 3D should appear as shown in this correction. Regretfully, we inadvertently used a transmission electron micrograph of an unrelated myophage from our laboratory archive in place of the correct micrograph of phage vB_SalP_SE29 during figure assembly.

Figure 3D legend: “(100.0K×; scale bar: 100 nm)” should read “(80.0K×; scale bar: 200 nm).”

We apologize for this error, which did not change the final result. The conclusions of the article remain intact.

**Fig 3:**